# Sexual function in primiparous women: a prospective study

**DOI:** 10.1007/s00192-021-05029-w

**Published:** 2022-01-01

**Authors:** Hedda Dahlgren, Markus H. Jansson, Karin Franzén, Ayako Hiyoshi, Kerstin Nilsson

**Affiliations:** 1 Department of Obstetrics and Gynecology, Örebro University Hospital, Region Örebro County, PO Box 1613, SE-701 16 Örebro, Sweden; 2 School of Medical Sciences, Faculty of Medicine and Health, Örebro University, Örebro, Sweden; 3 Clinical Epidemiology and Biostatistics, School of Medical Sciences, Örebro University, Örebro, Sweden

**Keywords:** Childbirth, Dyspareunia, PISQ-12, Postpartum, Sexual function

## Abstract

**Introduction and hypothesis:**

The aim of this prospective study was to examine the impact of sociodemographic, pregnancy and obstetric characteristics on sexual function 12 months postpartum in primiparous women. We hypothesized that sexual function would decrease after childbirth.

**Methods:**

Between 1 October 2014 and 1 October 2017, all nulliparous women in early pregnancy registering for maternity health care in Region Örebro County, Sweden, were invited to participate in this prospective study. A total of 958 women were included. Sexual activity and function were measured at early pregnancy, 8 weeks postpartum and 12 months postpartum using the Pelvic Organ Prolapse/Urinary Incontinence Sexual Questionnaire (PISQ-12). The associations between sociodemographic, pregnancy and obstetric characteristics and sexual activity and function from early pregnancy to 12 months postpartum were examined using linear and logistic models based on generalized estimating equations.

**Results:**

We found that the prevalence of sexually active women decreased from 98.0% in early pregnancy to 66.7% at 8 weeks postpartum, but increased to 90.0% at 12 months postpartum. Age ≥ 35 years, second-degree perineal tear and current breastfeeding were statistically significant risk factors for sexual inactivity at 12 months postpartum. Poor self-reported health in early pregnancy was statistically significantly associated with decreased sexual function at 12 months postpartum.

**Conclusions:**

A majority of women resumed sexual activity at 8 weeks postpartum and most women at 12 months postpartum; the decrease in sexual function at 12 months postpartum was small and few risk factors were observed.

## Introduction

Sexual dysfunction is common after delivery, with almost half of primiparous women reporting sexual dysfunction 12 months postpartum [1]. In addition, nulliparous women score better in sexual function than parous women [2]. Various components of childbirth have been studied as risk factors for both sexual dysfunction in general and specific aspects of sexual dysfunction. The major cause of decreased sexual function postpartum has not been established.

In a study by Anglès-Acedo in 2019, women with operative vaginal delivery were less likely to be sexually active at 6 months postpartum than women with vaginal delivery without operative assistance [3]. The importance of vaginal tears and obstetric anal sphincter injuries (OASI) is unclear. No difference in sexual satisfaction at 6 months postpartum was found among women with an intact perineum, second-degree perineal tear or OASI in one study [4], while another study described OASI as a risk factor for sexual inactivity at 6 months and for a lower sexual function score [3]. Furthermore, OASI has been found to be associated with less sexual activity after 1 year postpartum [5]. Women with urine and feces incontinence have been found to have poorer sexual function even several years after delivery [6]. Dyspareunia is common after delivery, although it decreases with time [7, 8], and has been suggested to be more common in women with OASI [9]. Other proposed risk factors for dyspareunia postpartum are pre-pregnancy dyspareunia [1] and breastfeeding [7, 10]. High maternal age has also been found to be a risk factor for sexual inactivity postpartum, while depressive symptoms have been suggested to affect sexual activity in the early postpartum period [11]. Cesarean delivery has not been proven to be protective of sexual function compared with operative vaginal delivery at 1 year postpartum [1], and sexual activity did not differ when comparing vaginal delivery, operative vaginal delivery and cesarean section [5]. Moreover, a study with a long follow-up, including postmenopausal women, found no difference in sexual function by delivery mode [2].

Information on sexual activity provided by healthcare professionals after delivery is mostly about contraceptives [12], even though sexual matters are a topic women want to discuss [7, 12]. Still, few women who experience sexual problems after delivery seek professional advice [7]. Knowledge of sexual function and sexual problems is crucial for healthcare professionals to be able to give adequate information. There is a lack of prospective studies that include pre-pregnancy data on sexual function. The aim of this study was to examine sexual function in early pregnancy and after delivery and to explore how sociodemographic, pregnancy and delivery-related characteristics affect sexual function at 12 months postpartum. We hypothesized that sexual function would decrease after delivery.

## Materials and methods

### Study design and population

From 1 October 2014 to 1 October 2017, nulliparous women registering for maternity health care in Region Örebro County, Sweden, were invited by their midwife to participate in the prospective observational study, POPRACT (Pelvic flOor in PRegnancy And ChildbirTh). Exclusion criteria were insufficient knowledge of Swedish and a gestational age > 16 weeks + 6 days. Web-based questionnaires were mailed to the women included in the study in early pregnancy, in late pregnancy (these data were not utilized in the present study), at 8 weeks postpartum and at 12 months postpartum. The questionnaires covered patients’ characteristics (i.e., education, weight, height and smoking habits), self-reported health, breastfeeding and whether they were sexually active or not. Sexually active women were asked about sexual dysfunction.

The results from the part of the POPRACT study on risk factors for perineal and vaginal tears were recently published [13].

### Outcome measures: Sexual activity and dysfunction

The primary outcome measures were sexual activity (“yes” or “no”) and sexual function, measured at early pregnancy and 12 months postpartum. The secondary outcome was sexual activity at 8 weeks postpartum.

Sexual function was measured using the Swedish translation of the Pelvic Organ Prolapse/Urinary Incontinence Sexual Questionnaire (PISQ-12) [14]. The PISQ-12 consists of 12 questions, asking the participants to consider their sexual function during the last 6 months, and was validated in 2003 [15]. The maximum score is 48 points; a higher score indicates better sexual function, and there is no cutoff value for dysfunction [15]. The PISQ-12 was scored taking into consideration the errata published by Rogers [16]. The following answers to the PISQ-12 sub-questions were dichotomized as “dysfunction”: the answers “seldom” and “never” for questions 1–4; the answers “always,” “usually” and “sometimes” for questions 5–11; the answers “much less intense” and “less intense” for question 12 (see Appendix Table 6). This categorization has previously been used for questions about pain [8, 9] and sexual desire [8].

When calculating each participant’s total PISQ-12 score, the mean value of the participant’s answered items was used to fill missing values in cases of up to two missing values. Questionnaires with more than two missing questions were excluded from the analysis of the total PISQ-12 score [15].

Since the PISQ-12 questions refer to the last 6 months, we analyzed PISQ-12 scores only at early pregnancy and at 12 months postpartum, and not for 8 weeks postpartum. Distributions of the answers to PISQ-12 from early pregnancy, 8 weeks and 12 months postpartum are presented in Appendix.

### Exposure measures

Obstetric variables were collected from a local laceration protocol or *Obstetrix*, a widely used obstetric journal system in Sweden. Use of the local laceration protocol has been validated [17]. A more detailed description of data collection from a local laceration protocol and from *Obstetrix* has been published [13].

The variables were categorized as follows: Delivery mode was grouped into: spontaneous vaginal, vaginal with vacuum extraction or cesarean section. Perineal tears were categorized in three groups: no laceration or first-degree laceration, second-degree laceration or OASI, in accordance with the Royal College of Obstetricians and Gynecologists classification [18]. Episiotomies were automatically recorded as a second-degree perineal tear, except in the case of OASI, when the perineal laceration diagnosis remained unchanged. Vaginal tears were categorized as either low or high, depending on whether the tear was limited to or extended above the distal third of the vagina, following the ICD-10 classification [19]. Self-reported health with a 5-point Likert scale was dichotomized as either good (“excellent,” “very good” and “good”) or poor (“fairly good” and “bad”). Body mass index (BMI) in early pregnancy was grouped into < 18.5, 18.5–24.9, 25.0–29.9 and > 30.0. Education was classified into elementary school, high school or university. Age was dichotomized at ≥ 35 years.

Data collected from the questionnaires were stored in the cloud-based tool esMaker 3.0 (Entergate AB, Sweden) in accordance with the General Data Protection Regulation of the European Union.

### Ethical consideration

Participation was voluntary, and exiting the study was possible at all times. Ethical approval was obtained from the Regional Ethical Review Board in Stockholm (registration number 2014/124–32).

### Statistical analyses

Descriptive data are presented as frequencies, proportions, means and standard deviations (SDs). Repeated measures of sexual activity, the PISQ-12 score and the PISQ-12 sub-questions were analyzed using generalized estimating equations (GEEs) to account for the correlation of data among individuals using exchangeable correlation structure. GEEs with linear models were used to obtain coefficients and 95% confidence intervals (CI) for the continuous outcomes of the total PISQ-12 score, and GEEs with logistic models were used to obtain the odds ratios (OR) for the binary outcomes of sexual activity and of the PISQ-12 sub-questions. Unadjusted and adjusted estimates were obtained, with the latter controlling for age, education, smoking, BMI, self-reported health, delivery mode, episiotomy, mode of the start of delivery, gestational age at delivery, degree of perineal tear, high vaginal tear and breastfeeding 12 months postpartum. All models included interactions between each variable and the categorical variable indicating time points. *P*-values < 0.05 were considered statistically significant. Data were analyzed using Stata/SE 16 (StataCorp LP, College Station, TX).

## Results

**Fig. 1:**
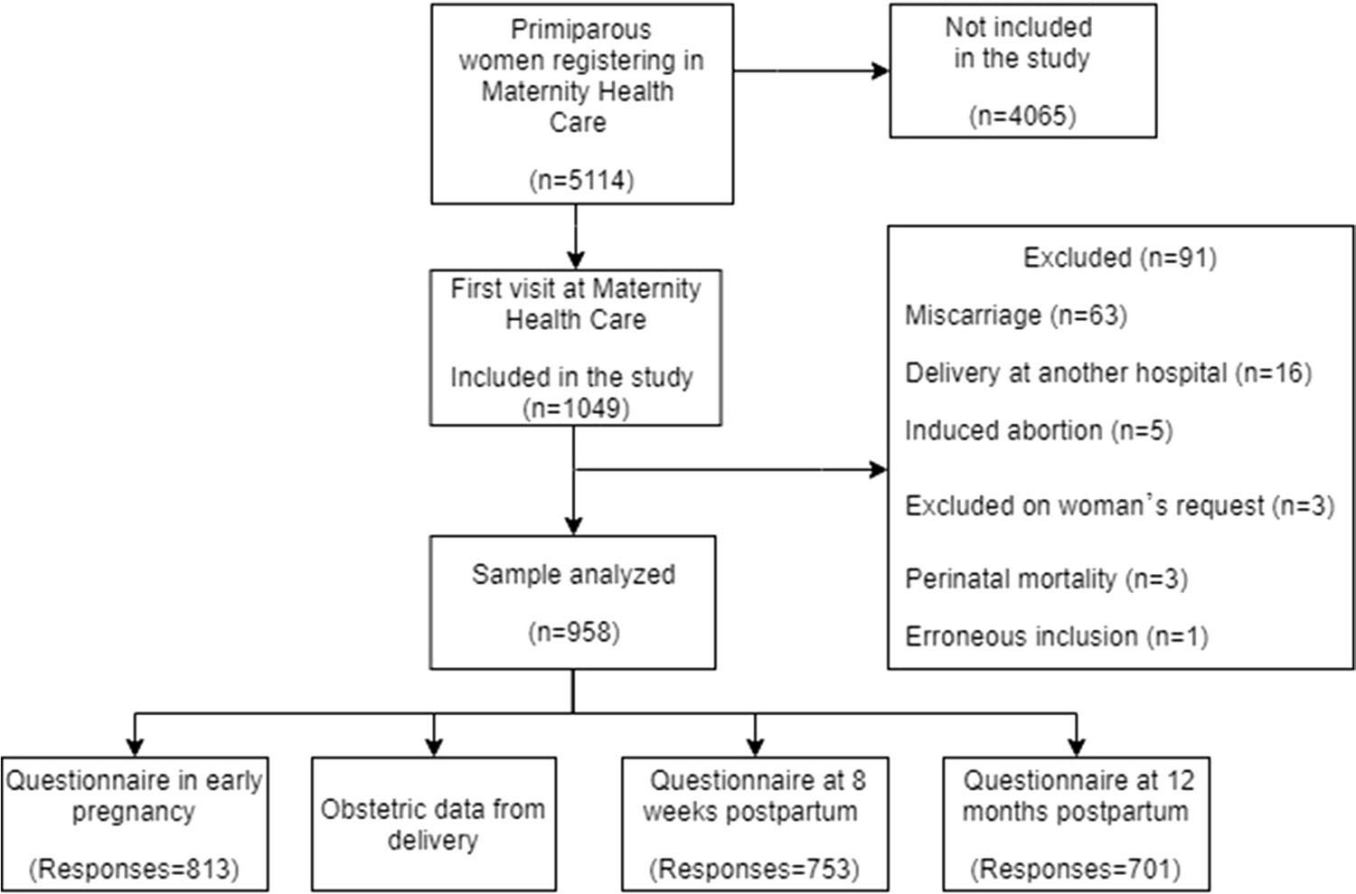
Flow chart illustrating the inclusion of the study sample

Figure 1 shows the inclusion and exclusion of women from early pregnancy to 12 months postpartum. The response rates were 77.5%, 71.8% and 66.8% for the questionnaires in early pregnancy, at 8 weeks and at 12 months postpartum, respectively. The mean (± SD) gestational age at questionnaire completion in early pregnancy was 11 weeks + 6 days (± 2 weeks + 4 days). The mean (±SD) time postpartum when the two questionnaires were completed after childbirth was 11 weeks + 2 days (± 2 weeks + 6 days) and 1 year + 3 weeks (± 3 weeks), respectively. One participant in early pregnancy and two participants in the postpartum questionnaires, respectively, were excluded for having more than two missing answers.

Tables 1 and 2 present the demographic and obstetric characteristics of the study participants. Most of the studied women were < 30 years of age, the majority had a university education, the great majority considered their health to be good or very good, and close to 90% delivered at term.

**Table 1:** Demographic characteristics of study participants in early pregnancy, at 8 weeks and at 12 months postpartum. Number and % of available data

Demographic characteristic	Early pregnancy	8 weeks postpartum	12 months postpartum
	N (%) Total: 958	N (%) Total: 753	N (%) Total: 701
Age, years			
< 25	126 (13.2)	90 (12.0)	79 (11.27)
25–29	454 (47.4)	362 (48.1)	338 (48.22)
30–34	295 (30.8)	238 (31.6)	225 (32.1)
≥ 35	83 (8.7)	63 (8.4)	59 (8.4)
Missing	0	0	0
Education			
Elementary school	14 (1.7)	10 (1.4)	9 (1.3)
High school	276 (34.0)	239 (33.1)	222 (32.9)
University	522 (64.3)	474 (65.6)	443 (65.7)
Missing	146	30	27
Smoking			
No	789 (97.5)	741 (98.8)	679 (96.9)
Yes	20 (2.5)	9 (1.2)	22 (3.1)
Missing	149	3	0
BMI in early pregnancy			
< 18.5	20 (2.5)	17 (2.4)	17 (2.5)
18.5–24.9	475 (58.7)	426 (59.1)	392 (58.3)
25.0–29.9	217 (26.8)	192 (26.3)	181 (26.9)
> 30.0	97 (12.0)	86 (11.9)	83 (12.3)
Missing	149	32	28
Self-reported health in early pregnancy			
Excellent	117 (14.4)	101 (13.9)	101 (14.9)
Very good	420 (51.5)	386 (53.2)	353 (52.1)
Good	255 (31.3)	223 (30.7)	208 (30.7)
Fairly good	21 (2.6)	15 (2.1)	13 (1.9)
Bad	2 (0.3)	1 (0.1)	2 (0.3)
Missing	143	27	24
Breastfeeding at 12 months postpartum			
No	.	.	563 (81.1)
Yes	.	.	131 (18.9)
Missing	.	.	7

**Table 2:** Obstetric characteristics of study participants. Number and % of available data

Obstetric characteristic	**N** (%) Total: 958
Gestational age at delivery (w + d)	
Mean (SD), range	39w + 6d (2w), 26w + 5d–42w + 5d
Pre-term (< 37 + 0)	54 (5.8)
Term (37 + 0 to 42 + 0)	830 (88.6)
Post-term (> 42 + 0)	53 (5.7)
Missing	21
Delivery mode	
Spontaneous vaginally	658 (69.0)
Vaginal with vacuum extraction	148 (15.5)
Cesarean section	148 (15.5)
Missing	4
Delivery start	
Spontaneous	702 (73.7)
Induction vaginal delivery	195 (20.5)
Cesarean section before labor	55 (5.8)
Missing	6
Episiotomy	
No	584 (91.1)
Yes	57 (8.9)
Missing	317
Number of births	
Singleton	942 (99.1)
Twins	9 (1.0)
Missing	7
Degree of perineal tear	
No injury or first degree	447 (57.7)
Second degree	282 (36.4)
Obstetric anal sphincter injury	46 (5.9)
Missing	183
High vaginal tear	
No	671 (88.6)
Yes	86 (11.4)
Missing	201

### Sexual activity

In early pregnancy, 797 (98.0%) of the participants were sexually active; this number decreased to 502 (66.7%) at 8 weeks postpartum and then rose to 631 (90.0%) at 12 months postpartum (see Appendix Table 6). Table 3 presents the associations between demographic and obstetric factors and sexual inactivity. In the adjusted model, the only statistically significant associations with sexual inactivity in early pregnancy were age ≥ 35 years and poor self-reported health compared with good health. Second-degree perineal tear, OASI and current breastfeeding were associated with a statistically significant increased risk for sexual inactivity at 8 weeks postpartum. At 12 months postpartum, second-degree perineal tear, current breastfeeding and age ≥ 35 years were statistically significantly associated with higher risk of sexual inactivity. High school education, compared with elementary school education, showed a statistically significant raised risk for sexual inactivity at 12 months, but not in early pregnancy or at 8 weeks postpartum. Compared with non-smokers, smokers showed a lower likelihood of sexual inactivity in early pregnancy and at 12 months postpartum.

**Table 3:** Odds ratios and 95% confidence intervals for risk of sexual inactivity from early pregnancy to 12 months postpartum

	OR (95% CI)				aOR (95% CI)			
	N	Early pregnancy	8 weeks postpartum	12 months postpartum	*N* = 517	Early pregnancy	8 weeks postpartum	12 months postpartum
Age	847							
< 35		Ref	Ref	Ref		Ref	Ref	Ref
≥ 35		7.23 (2.55, 20.47)*	1.92 (1.14, 3.22)*	4.59 (2.46, 8.59)*		28.95 (4.37, 191.58)*	1.80 (0.88, 3.67)	5.57 (2.38, 13.05)*
Education	811							
Elementary school		1.29 (0.15, 10.76)	0.19 (0.02, 1.53)	1.29 (0.15, 10.76)		3.28 (0.32, 33.21)	0.42 (0.04, 4.56)	3.28 (0.32, 33.21)
High school		1.93 (0.71, 5.19)	0.70 (0.50, 0.98)*	1.56 (0.93, 2.60)		3.86 (0.83, 17.90)	1.20 (0.79, 1.84)	2.20 (1.16, 4.15)*
University		Ref	Ref	Ref		Ref	Ref	Ref
Smoking	808							
No		Ref	Ref	Ref		Ref	Ref	Ref
Yes		0.16 (0.03, 0.77)*	2.40 (0.67, 8.66)	0.30 (0.09, 0.98)*		0.02 (0.00, 0.24)*	1.49 (0.36, 6.10)	0.23 (0.06, 0.88)*
BMI in early pregnancy	806							
< 18.5		1.25 (0.27, 5.80)	1.32 (0.49, 3.54)	1.25 (0.27, 5.80)		0.71 (0.13, 3.95)	1.24 (0.38, 4.09)	0.71 (0.13, 3.95)
18.5–24.9		Ref	Ref	Ref		Ref	Ref	Ref
25.0–29.9		0.59 (0.16, 2.15)	0.91 (0.63, 1.31)	1.19 (0.67, 2.11)		0.55 (0.10, 3.09)	0.98 (0.62, 1.53)	1.08 (0.54, 2.16)
> 30.0		0.89 (0.19, 4.07)	0.85 (0.52, 1.40)	1.27 (0.60, 2.69)		0.11 (0.01, 1.76)	0.63 (0.33, 1.20)	1.03 (0.40, 2.64)
Self-reported health in early pregnancy	814							
Good		Ref	Ref	Ref		Ref	Ref	Ref
Poor		13.67 (4.04, 46.31)*	0.90 (0.31, 2.59)	3.83 (1.23, 11.89)*		18.81 (1.98, 178.60)*	0.76 (0.19, 2.98)	3.52 (0.85, 14.53)
Delivery mode	843							
Vaginal		Ref	Ref	Ref		Ref	Ref	Ref
Vaginal With vacuum extraction		1.94 (0.59, 6.37)	1.26 (0.84, 1.90)	0.91 (0.45, 1.83)		1.75 (0.30, 10.38)	0.93 (0.53, 1.64)	1.21 (0.52, 2.82)
All Cesarean section		1.44 (0.38, 5.46)	0.71 (0.45, 1.13)	0.92 (0.44, 1.90)		0.54 (0.03, 10.79)	1.05 (0.57, 1.92)	0.68 (0.25, 1.86)
Gestational age at delivery	829							
< 37 + 0		2.42 (0.53, 11.07)	0.54 (0.25, 1.18)	0.87 (0.27, 2.79)		4.89 (0.30, 79.62)	0.67 (0.23, 1.91)	2.05 (0.51, 8.27)
37 + 0 to 42 + 0		Ref	Ref	Ref		Ref	Ref	Ref
> 42 + 0		1.28 (0.17, 9.68)	1.67 (0.90, 3.10)	0.94 (0.32, 2.72)		1.99 (0.17, 23.31)	0.82 (0.35, 1.88)	0.89 (0.25, 3.12)
Degree perineal tear	682							
No injury or first degree		Ref	Ref	Ref		Ref	Ref	Ref
Second degree		2.66 (0.86, 8.23)	2.09 (1.47, 2.98)*	1.57 (0.89, 2.75)		5.28 (0.66, 42.39)	2.23 (1.41, 3.51)*	2.20 (1.06, 4.58)*
OASI		1.90 (0.67, 5.40)	6.18 (2.83, 13.47)*	1.90 (0.67, 5.40)		1.94 (0.51, 7.42)	6.77 (2.67, 17.20)*	1.94 (0.51, 7.42)
High vaginal tear	669							
No		Ref	Ref	Ref		Ref	Ref	Ref
Yes		2.28 (0.61, 8.50)	2.08 (1.26, 3.44)*	1.17 (0.52, 2.59)		6.06 (0.95, 38.53)	1.38 (0.75, 2.53)	1.04 (0.41, 2.60)
Breastfeeding at 12 months postpartum	697							
No		Ref	Ref	Ref		Ref	Ref	Ref
Yes		1.30 (0.35, 4.78)	1.62 (1.09, 2.42)*	3.13 (1.85, 5.30)*		1.38 (0.23, 8.29)	1.80 (1.10, 2.95)*	4.36 (2.26, 8.41)*

Generalized estimating equations with a logistic model were used to calculate odds ratios (OR)

Adjusted ORs were obtained using a model including age, education, smoking, BMI, self-reported health in early pregnancy, delivery mode, gestational age at delivery, degree of perineal tear, high vaginal tear and breastfeeding 12 months postpartum

*Significant

OASI = obstetric anal sphincter injury

### Sexual function measured as PISQ-12 score

Table 6 in the Appendix presents the frequency of answers to each sub-question, and Table A2 presents the change in dysfunction for each sub-question. Table 4 presents the mean and SD for the total PISQ-12 score and the difference in the total PISQ-12 score at early pregnancy and at 12 months postpartum by exposure measures. In early pregnancy, a statistically significant difference in lower sexual function was observed for poor self-reported health; this difference remained significant at 12 months postpartum and after adjustments were made for other variables. At 12 months postpartum, in the adjusted model, the difference in sexual function as associated with OASI compared with no or first-degree perineal tear became greater than in early pregnancy, although the association was not statistically significant.

**Table 4:** Mean and coefficients for total PISQ-12 score with 95% confidence intervals at early pregnancy and 12 months postpartum

			Unadjusted model		Adjusted model	
	Early pregnancy	12 months postpartum	Early pregnancy	12 months postpartum	Early pregnancy	12 months postpartum
	Mean (SD)	Mean (SD)	Coefficient (95% CI)	Coefficient (95% CI)	Coefficient (95% CI)	Coefficient (95% CI)
All	40.3 (3.3)	38.4 (4.3)				
Age						
< 35	40.2 (3.3)	38.4 (4.3)	Ref	(Ref)	(Ref)	(Ref)
≥35	40.5 (3.4)	38.4 (4.9)	0.36 (−0.61, 1.34)	−0.35 (−1.53, 0.82)	−0.08 (−1.70, 1.54)	0.22 (−1.62, 2.05)
Education						
Elementary school	39.7 (2.7)	38.9 (3.3)	−0.61 (−2.61, 1.40)	0.25 (−2.20, 2.70)	0.04 (−3.41, 3.49)	1.57 (−2.09, 5.23)
High school	40.1 (3.4)	38.1 (4.7)	−0.19 (−0.74, 0.37)	−0.39 (−1.02, 0.24)	−0.33 (−1.12, 0.45)	−0.27 (−1.11, 0.56)
University	40.3 (3.3)	38.5 (4.1)	(Ref)	(Ref)	(Ref)	(Ref)
Smoking						
No	40.3 (3.3)	38.4 (4.3)	(Ref)	(Ref)	(Ref)	(Ref)
Yes	39.7 (3.7)	37 (3.5)	−0.59 (−2.36, 1.18)	−1.11 (−3.50, 1.29)	−0.49 (−3.24, 2.25)	−0.65 (−3.83, 2.53)
BMI in early pregnancy						
< 18.5	40.5 (3.7)	38 (5.0)	0.39 (−1.30, 2.08)	−0.02 (−1.90, 1.85)	−2.20 (−4.75, 0.35)	−1.56 (−4.32, 1.21)
18.5–24.9	40.1 (3.3)	38.3 (4.2)	(Ref)	(Ref)	(Ref)	(Ref)
25.0–29.9	40.4 (3.3)	38.4 (4.6)	0.31 (−0.30, 0.93)	−0.01 (−0.69, 0.68)	−0.28 (−1.11, 0.55)	0.23 (−0.68, 1.13)
> 30.0	40.6 (3.2)	38.7 (4.4)	0.46 (−0.38, 1.29)	0.37 (−0.56, 1.29)	−0.37 (−1.48, 0.73)	0.48 (−0.73, 1.69)
Self-reported health in early pregnancy						
Good	40.3 (3.3)	38.5 (4.3)	(Ref)	(Ref)	(Ref)	(Ref)
Poor	37.9 (3.5)	35 (2.6)	−2.36 (−4.07, −0.65)*	−3.29 (−5.37, −1.20)*	−2.67 (−5.27, −0.07)*	−5.85 (−8.58, −3.13)*
Delivery mode						
Vaginal	40.3 (3.4)	38.4 (4.3)	(Ref)	(Ref)	(Ref)	(Ref)
Vaginal with vacuum extraction	40.3 (3.7)	38.4 (4.4)	0.05 (−0.68, 0.79)	−0.26 (−1.07, 0.55)	−0.01 (−1.00, 0.98)	−0.53 (−4.22, 3.16)
All cesarean section	40.2 (3.4)	38.3 (4.4)	−0.10 (−0.85, 0.64)	−0.24 (−1.07, 0.59)	−0.62 (−4.30, 3.07)	0.10 (−0.95, 1.14)
Episiotomy						
No	40.2 (3.3)	38.6 (4.3)	(Ref)	(Ref)	(Ref)	(Ref)
Yes	40.1 (2.9)	38 (3.2)	−0.03 (−1.12, 1.07)	−0.80 (−2.03, 0.42)	0.32 (−1.11, 1.76)	−0.55 (−2.03, 0.93)
Start of delivery						
Spontaneous vaginal	40.2 (3.3)	38.4 (4.3)	(Ref)	(Ref)	(Ref)	(Ref)
Induction	40.5 (3.3)	38.6 (4.5)	0.31 (−0.35, 0.97)	0.27 (−0.46, 1.00)	0.90 (−0.14, 1.94)	0.69 (−0.39, 1.78)
Cesarean section before labor	39.9 (3.6)	38.1 (4.6)	−0.34 (−1.49, 0.82)	−0.68 (−1.96, 0.60)	NE	NE
Gestational age at delivery						
< 37 + 0	40.2 (2.4)	38.8 (4.3)	−0.07 (−1.22, 1.09)	0.46 (−0.82, 1.74)	0.29 (−2.17, 2.74)	2.24 (−0.22, 4.70)
37 + 0 to 42 + 0	40.3 (3.4)	38.4 (4.3)	(Ref)	(Ref)	(Ref)	(Ref)
> 42 + 0	40.0 (3.7)	38.1 (5.8)	−0.22 (−1.39, 0.94)	−0.09 (−1.36, 1.18)	−0.63 (−2.41, 1.15)	0.44 (−1.42, 2.30)
Degree perineal tear						
No injury or first degree	40.2 (3.4)	38.5 (4.4)	(Ref)	(Ref)	(Ref)	(Ref)
Second degree	40.3 (3.2)	38.5 (4.0)	0.13 (−0.49, 0.74)	−0.06 (−0.74, 0.62)	0.04 (−0.75, 0.84)	−0.28 (−1.13, 0.56)
OASI	39.6 (3.9)	37.8 (4.5)	−0.46 (−1.76, 0.84)	−0.70 (−2.16, 0.76)	−0.82 (−2.35, 0.70)	−1.61 (−3.21, 0.00)
High vaginal tear						
No	40.3 (3.4)	38.6 (4.3)	(Ref)	(Ref)	(Ref)	(Ref)
Yes	39.9 (3.0)	38.6 (4.1)	−0.40 (−1.32, 0.52)	−0.25 (−1.25, 0.74)	0.13 (−0.95, 1.20)	0.56 (−0.57, 1.68)
Breastfeeding at 12 months postpartum						
No	40.3 (3.2)	38.6 (4.2)	(Ref)	(Ref)	(Ref)	(Ref)
Yes	39.9 (3.7)	37.6 (4.8)	−0.39 (−1.14, 0.36)	−1.06 (−1.86, −0.25)*	−0.42 (−1.38, 0.55)	−0.24 (−1.31, 0.82)

Maximum total score is 48.0 points

Generalized estimating equations with a linear model were used to estimate differences in score (coefficients)

Adjusted model included age, education, smoking, BMI, self-reported health in early pregnancy, delivery mode, episiotomy, start of delivery, gestational age at delivery, degree of perineal tear, high vaginal tear and breastfeeding 12 months postpartum

*Significant

NE = not estimated

OASI = obstetric anal sphincter injury

### Sub-scores: Satisfaction

For question 4 in the questionnaire, “Are you satisfied with the variety of sexual activities?,” poor self-reported health in early pregnancy was the only statistically significant risk factor for being unsatisfied with the variety of sexual activities at 12 months postpartum, when adjusted for smoking, self-reported health in early pregnancy, delivery mode and degree of perineal tear (data not shown).

### Sub-scores: Dyspareunia

In early pregnancy, 18.2% of the women suffered from dyspareunia, as measured by question 5; at 12 months postpartum, this had increased to 29.8% (see Appendix Table 6). Table 5 presents risk factors for dyspareunia. After adjustment, a statistically significant higher risk for dyspareunia was observed for second-degree perineal tear and OASI compared with women without injury or with first-degree tear, respectively. Compared with vaginal delivery without vacuum extraction, delivery with vacuum extraction showed a statistically significant association in the unadjusted model; however, after the adjustment, the odds ratio (OR) declined and this association was no longer statistically significant. To explore whether this change was due to an over-adjustment of other factors naturally associated with vacuum extraction, an alternative adjusted analysis was performed that excluded perineal tear, high vaginal tear, episiotomy and the mode of delivery start. In this alternative model, vacuum extraction remained significant at 12 months postpartum, with an adjusted OR of 1.63 (95% CI 1.01, 2.63).

**Table 5:** Odds ratios and 95% confidence intervals for risk of dyspareunia

	OR (95% CI)			aOR (95% CI)		
	N = varies	Early pregnancy	12 months postpartum	N = 418	Early pregnancy	12 months postpartum
Age	822					
< 35		Ref	Ref		Ref	Ref
≥35		1.07 (0.56, 2.07)	1.37 (0.71, 2.65)		1.77 (0.63, 4.96)	1.87 (0.60, 5.82)
Education	798					
Elementary school		0.84 (0.18, 3.80)	1.65 (0.40, 6.78)		1.78 (0.16, 20.13)	1.15 (0.12, 10.96)
High school		1.40 (0.96, 2.02)	1.42 (0.99, 2.04)		1.34 (0.77, 2.34)	1.24 (0.74, 2.08)
University		Ref	Ref		Ref	Ref
Smoking	795					
No		Ref	Ref		Ref	Ref
Yes		1.05 (0.31, 3.60)	0.60 (0.17, 2.07)		1.21 (0.15, 10.06)	0.79 (0.14, 4.36)
BMI in early pregnancy	795					
< 18.5		1.95 (0.73, 5.23)	2.50 (0.90, 6.98)		1.32 (0.24, 7.32)	2.47 (0.50, 12.24)
18.5–24.9		Ref	Ref		Ref	Ref
25.0–29.9		1.09 (0.72, 1.65)	0.79 (0.53, 1.20)		1.34 (0.73, 2.46)	0.70 (0.38, 1.27)
> 30.0		0.79 (0.43, 1.46)	0.93 (0.54, 1.59)		0.80 (0.33, 1.97)	1.08 (0.53, 2.22)
Self-reported health in early pregnancy	801					
Good		Ref	Ref		Ref	Ref
Poor		5.27 (2.10, 13.21)*	3.50 (1.06, 11.55)*		1.87 (0.35, 9.97)	3.20 (0.68, 15.05)
Delivery mode	818					
Vaginal		Ref	Ref		Ref	Ref
Vacuum extraction		1.19 (0.73, 1.93)	1.86 (1.19, 2.89)*		1.00 (0.48, 2.08)	1.46 (0.79, 2.68)
All cesarean section		1.08 (0.66, 1.79)	1.37 (0.86, 2.21)		1.49 (0.14, 15.75)	3.98 (0.51, 30.76)
Start of delivery	816					
Spontaneous		Ref	Ref		Ref	Ref
Induction		0.93 (0.59, 1.48)	1.21 (0.80, 1.83)		0.58 (0.25, 1.34)	1.00 (0.51, 1.98)
Cesarean section before labor		1.30 (0.63, 2.72)	0.92 (0.42, 2.01)		NE	NE
Episiotomy	553					
No		Ref	Ref		Ref	Ref
Yes		1.34 (0.66, 2.74)	1.45 (0.70, 2.97)		1.77 (0.64, 4.87)	0.85 (0.35, 2.06)
Gestational age at delivery	803					
< 37 + 0		1.03 (0.47, 2.28)	0.59 (0.26, 1.38)		1.39 (0.27, 7.03)	1.39 (0.27, 7.03)
37 + 0 to 42 + 0		Ref	Ref		Ref	Ref
> 42 + 0		2.05 (1.04, 4.02)*	1.29 (0.65, 2.55)		3.35 (1.03, 10.87)*	1.22 (0.39, 3.78)
Degree of perineal tear	663					
No injury or first degree		Ref	Ref		Ref	Ref
Second degree		0.75 (0.48, 1.16)	1.48 (0.99, 2.20)		0.59 (0.32, 1.09)	1.69 (1.00, 2.88)*
OASI		1.16 (0.51, 2.68)	3.19 (1.47, 6.93)*		0.92 (0.32, 2.65)	4.32 (1.71, 10.88)*
High vaginal tear	650					
No		Ref	Ref		Ref	Ref
Yes		1.34 (0.74, 2.43)	1.96 (1.14, 3.38)*		1.22 (0.56, 2.65)	1.07 (0.55, 2.08)
Breastfeeding at 12 months postpartum	685					
No		Ref	Ref		Ref	Ref
Yes		1.44 (0.89, 2.32)	1.09 (0.69, 1.71)		1.33 (0.69, 2.56)	0.95 (0.48, 1.86)

Dyspareunia was defined based on the answers “always,” “usually” or “sometimes” for the sub-question of PISQ-12: “Do you feel pain during sexual intercourse?”

Generalized estimating equations with a logistic model were used to calculate odds ratios (OR)

Adjusted ORs were obtained using a model including for age, education, smoking, BMI, self-reported health, delivery mode, start of delivery, episiotomy, gestational age at delivery, degree of perineal tear, high vaginal tear and breastfeeding 12 months postpartum

*Significant

NE = not estimated

OASI = obstetric anal sphincter injury

## Discussion

In this prospective study of primiparous women, we found that sexual activity decreased from 98.0% in early pregnancy to 66.7% at 8 weeks postpartum, but increased to 90.0% at 12 months postpartum. Significant risk factors for sexual inactivity at 12 months postpartum were age ≥ 35 years, second-degree perineal tear, current breastfeeding and a high school education, whereas smoking was associated with sexual activity. Poor self-reported health in early pregnancy was a statistically significant risk factor for decreased PISQ-12 total score at 12 months postpartum.

### Sexual activity

Our finding that the great majority of studied women resumed sexual activity after childbirth within 12 months postpartum is similar to other findings [11, 20]. Notably, we did not find the mode of delivery [21] or episiotomy [8, 11] to affect sexual activity at 12 months postpartum. We found that second-degree perineal tear and OASI were associated with less sexual activity at 8 weeks postpartum, although only second-degree perineal tear remained significant at 12 months postpartum. Our results support previous findings that OASI is associated with less sexual activity at 6 months postpartum. Anglès-Acedo found that 98% of women with spontaneous vaginal delivery without OASI are sexually active, in contrast to 77% of women with OASI [3]. In addition, Brubaker found that 94% of women without OASI were sexually active compared with 88% of women with OASI [8]. Rådestad et al. found OASI to extend the time to first sexual intercourse; however, as in our study, no difference was seen at 12 months postpartum, at which point 96% of the studied women had been sexually active [11]. Similar to our results at 12 months postpartum, Leeman et al. found that the great majority of studied women, 87%, resumed sexual activity at 6 months postpartum with no difference between tear groups. Leeman et al. combined second-degree perineal tear and OASI when analyzing [22]. Buhling et al. investigated the time to first sexual intercourse until 8 weeks postpartum and found no difference among delivery mode or the degree of perineal tear; however, their data consisted of deliveries taking place in 1997–1999 [21], and diagnostic procedures and the handling of tears might have been different at that time.

We found higher maternal age to affect the time taken to resume sexual activity, consistent with Rådestad et al. who found that an age > 35 years was a risk factor for being sexually inactive at 12 months postpartum [11]. An age of 36 years or older has been described as a risk factor for waiting an average of 2.5 weeks longer to resume sexual activity compared with an age < 25 years [4]. In our study, we only obtained data on sexual activity at two time points after childbirth, namely, at 8 weeks and 12 months postpartum. In contrast, Brubaker et al. dichotomized age at 30 years and found no age difference in the resumption of sexual activity at 6 months postpartum [8].

Our finding that a high school education was associated with sexual inactivity at 12 months postpartum is somewhat surprising and is inconsistent with previous studies; Brubaker et al. found no association between level of education and sexual inactivity [8].

### Sexual function

In general, the decrease in sexual function from early pregnancy to 12 months postpartum was small. Poor self-reported health in early pregnancy was found to be the only statistically significant risk factor for a decreased total PISQ-12 score at 12 months postpartum. Consistent with our finding, Brubaker et al. studied the PISQ-12 score at 6 months postpartum and did not find age, dichotomized at 30 years, to affect the total PISQ-12 score, although their mean value (scoring 39 ± 4) was similar to ours [8].

Unlike our expectation that OASI would be a risk factor for decreased sexual function postpartum, the association between OASI and decreased sexual function did not reach statistical significance. Two smaller studies at 6 months postpartum also found no association between OASI and the total PISQ-12 score in primiparous women [8] or in a mixed group of primiparous and multiparas women [3]. Furthermore, we did not find delivery mode to affect sexual function, which aligns with previous findings [1, 23]. Current breastfeeding had no impact on the total PISQ-12 score in our study, whereas Anglès-Acedo et al. found a negative association at 6 months postpartum. However, their study was not restricted to primiparous women [3].

Question 4 regarding satisfaction with the variety of sexual activities was difficult to analyze because few women scored for dysfunction. It is unclear whether the participants answered strictly about their satisfaction with the variety of activities (i.e., different types of sex) or whether their answers referred more generally to their satisfaction with their sexual life. We think that information about the latter would be of even greater importance. Olsson et al. arranged focus group discussions with women after childbirth and found that women wanted to sleep and have time for themselves, as well as not feeling comfortable with their body changes after pregnancy and delivery [12]. Interviews with fathers post-delivery also found that sleep had a higher priority than sex and that the fathers considered general closeness and caring as a part of their current sexuality [24].

Our finding that dyspareunia affects 30% of primiparous women at 12 months postpartum aligns with the results from previous studies [7, 8]. Statistically significant risk factors were second-degree perineal tear and OASI, which aligns with the findings of Leeman et al., who found perineal tear or OASI to be a statistically significant risk factor for dyspareunia 6 months postpartum [22]. Others have also found evidence of dyspareunia due to OASI [21, 22].

Our findings show overall good sexual function, and responses indicating sexual dysfunction were rare in each sub-question. Poor self-reported health in early pregnancy was a statistically significant risk factor for sexual inactivity in early pregnancy and for scoring lower in the total PISQ-12 in early pregnancy. Moreover, health in early pregnancy affected sexual function at 12 months postpartum, but did not affect the probability of being sexually active postpartum. To the best of our knowledge, there is little research on self-reported health and sexual function during pregnancy and after childbirth. Schytt et al. found poor self-reported health to improve during pregnancy, decline 2 months after childbirth and increase again at 1 year postpartum. Increased poor self-reported health was also seen in the partners at 1 year post-delivery [25]. Stepanikova et al. found that good self-reported health in pregnancy correlated with fewer maternal health problems at 6 months, 18 months and even 3 years after childbirth [26]. Our findings might reveal some of the complex origins of sexual function and its interplay with the psychological aspect, extending beyond this study. It is not well understood how long obstetric outcomes influence sexual function beyond 1 year. We suggest further research on sexual function postpartum, preferably considering new parents’ expectations of alteration of sexual activity after childbirth.

### Strengths and limitations

The strength of this prospective study is the availability of early pregnancy data, which were unavailable or collected retrospectively in previous studies [8, 9], increasing the risk of recall bias. Furthermore, limiting the sample to primiparous women avoided the risk of impact from previous deliveries.

The PISQ-12 questions were administered only to women who answered “yes” to the question “Are you sexually active?” There was no definition of what “sexually active” might entail, so the woman’s own interpretation of sexual activity was decisive. Since being sexually active is a subjective judgment we believe this to be appropriate. The International Urogynecological Association (IUGA) modified the PISQ score and in 2013 validated the PISQ IUGA-Revised (PISQ-IR). The use of PISQ-IR would have given us more complete information about sexual activity and reasons not to be sexually active and there would be the benefit of gender-neutral questions [27], but as far as we know a Swedish translation of the PISQ-IR has not been validated.

Since the PISQ-12 requests participants to consider the last 6 months, we analyzed PISQ-12 scoring at the early pregnancy and 12 months postpartum, not including 8 weeks postpartum, since answers from this period would mainly reflect the late pregnancy period, which was not the focus of our study. The PISQ-12 is validated for heterosexual women with a partner [15]; however, as far as we know, it has not been validated for pregnant women. Although aware of this, we used the questionnaire for women in early pregnancy, as the questionnaire characteristics of pelvic floor dysfunction suited the overall project (the POPRACT study) well. It is possible that women in early pregnancy might suffer from, for example, nausea and tiredness affecting sexual function, although the participants were asked to consider the past 6 months when answering. Another limitation might be the inclusion criteria of understanding Swedish; we might have missed cultural differences in sexuality and expectations of life after childbirth.

It is not clear whether findings of dysfunction 12 months postpartum could be considered irreversible, although tissue healing and remodeling has probably stopped. However, psychological, social and partner-related factors certainly continue to change beyond 1 year after childbirth.

We calculated PISQ-12 scoring by the errata [16] published in 2004. Some recent studies [9] have calculated based on the original PISQ-12 validation [15] published in 2003; consequently, it is crucial that studies clearly present their methods when using the PISQ-12.

## Conclusion

In this prospective longitudinal study of primiparous women, we found few statistically significant risk factors for sexual inactivity at 8 weeks and 12 months postpartum. The decrease in the total PISQ-12 score from early pregnancy to 12 months postpartum showed little association with risk factors. These results are important to communicate to women and healthcare providers, since giving birth can result in major stress and worry. This information can be used to reassure women that certain childbirth factors and obstetric outcomes do not per se result in sexual dysfunction. Moreover, problems experienced in the early postpartum period can decrease or disappear in the longer term. Since postpartum women request discussion on sexual matters from healthcare workers, it is important to meet such requests with evidence-based advice.

## Figures and Tables

**Fig. 2:**
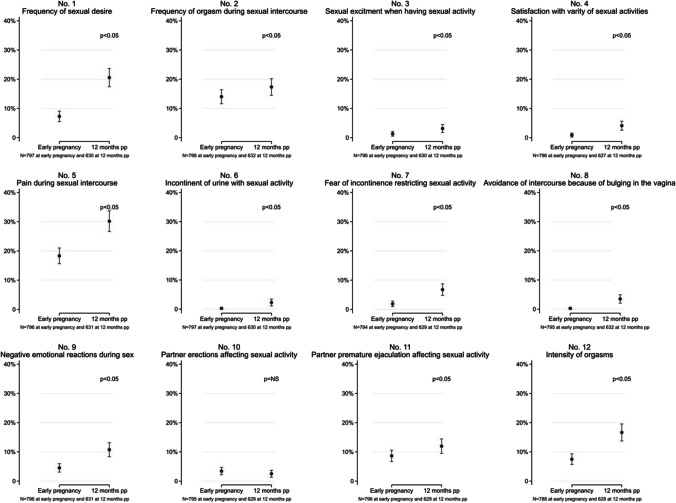
Frequency of sexual dysfunction in early pregnancy and 12 months postpartum. Change in prevalence of sexual dysfunction from early pregnancy to 12 months postpartum for each PISQ-12 sub-question. Total number varies due to missing data. Answers to PISQ-12 sub-questions were dichotomized as dysfunction for questions 1–4 when answering “seldom” and “never;” questions 5–11 were dichotomized as dysfunction when answering “always,” “usually” and “sometimes;” question 12 was considered dysfunction for “much less intense” and “less intense.” *P* values were obtained from unadjusted logistic models using generalized estimating equations. NS = non-significant

## Appendix

Table 6 and Fig. 2

**Table 6:** Frequency of sexual activity and answers to each PISQ-12 question in early pregnancy, at 8 weeks postpartum and 12 months postpartum

No.	Question	Early pregnancy	8 weeks postpartum	12 months postpartum
		N (%)	N (%)	N (%)
	Are you sexually active?			
	Total	813	753	701
	Yes	797 (98.0)	502 (66.7)	631 (90.0)
	No	16 (2.0)	252 (33.5)	70 (10.0)
1	How frequently do you feel sexual desire?			
	Total	797	502	630
	Always	11 (1.4)	9 (1.8)	8 (1.3)
	Usually	340 (42.7)	144 (28.7)	148 (23.5)
	Sometimes	388 (48.7)	253 (50.4)	348 (55.2)
	Seldom	55 (6.9)	93 (18.5)	118 (18.7)
	Never	3 (0.4)	3 (0.6)	8 (1.3)
2	Do you climax (have an orgasm) when having sexual intercourse with your partner?			
	Total	797	502	631
	Always	194 (24.3)	110 (21.8)	151 (23.9)
	Usually	328 (41.2)	173 (34.4)	227 (36.0)
	Sometimes	164 (20.6)	139 (27.7)	146 (23.1)
	Seldom	66 (8.3)	50 (9.9)	65 (10.3)
	Never	45 (5.6)	30 (6.0)	42 (6.7)
3	Do you feel sexually excited (turned on) when having sexual activity with your partner?			
	Total	796	502	630
	Always	446 (56.0)	224 (44.6)	248 (39.4)
	Usually	287 (36.1)	198 (39.3)	268 (42.5)
	Sometimes	53 (6.7)	68 (13.5)	94 (14.9)
	Seldom	10 (1.3)	10 (2.0)	17 (2.7)
	Never	.	2 (0.4)	3 (0.5)
4	How satisfied are you with the variety of sexual activities in your current sex life?			
	Total	796	502	627
	Always	353 (44.4)	164 (32.6)	175 (27.9)
	Usually	360 (45.2)	225 (44.8)	293 (46.7)
	Sometimes	76 (9.6)	86 (17.1)	134 (21.4)
	Seldom	7 (0.9)	25 (5.0)	23 (3.7)
	Never	.	2 (0.4)	2 (0.3)
5	Do you feel pain during sexual intercourse?			
	Total	796	502	631
	Always	5 (0.6)	8 (1.6)	7 (1.1)
	Usually	13 (1.6)	23 (4.6)	36 (5.7)
	Sometimes	127 (16.0)	123 (24.4)	145 (23.0)
	Seldom	354 (44.5)	219 (43.76	229 (36.3)
	Never	297 (37.3)	129 (25.6)	214 (33.9)
6	Are you incontinent of urine (leak urine) with sexual activity?			
	Total	797	502	630
	Always	.	.	.
	Usually	.	.	4 (0.6)
	Sometimes	2 (0.3)	12 (2.4)	10 (1.6)
	Seldom	28 (3.5)	47 (9.3)	54 (8.6)
	Never	767 (96.2)	443 (88.2)	562 (89.2)
7	Does fear of incontinence (either stool or urine) restrict your sexual activity?			
	Total	794	502	629
	Always	.	2 (0.4)	3 (0.5)
	Usually	2 (0.3)	8 (1.6)	6 (1.0)
	Sometimes	13 (1.6)	24 (4.8)	34 (5.4)
	Seldom	36 (4.5)	40 (8.0)	53 (8.4)
	Never	743 (93.6)	428 (85.3)	533 (84.7)
8	Do you avoid sexual intercourse because of bulging in the vagina (either the bladder, rectum, or vagina falling out)?			
	Total	795	502	631
	Always	1 (0.1)	1 (0.2)	1 (0.2)
	Usually	.	5 (1.0)	5 (0.8)
	Sometimes	1 (0.1)	9 (1.8)	16 (2.5)
	Seldom	3 (0.4)	11 (2.2)	15 (2.4)
	Never	790 (99.4)	476 (94.8)	594 (94.1)
9	When you have sex with your partner, do you have negative emotional reactions such as fear, disgust, shame, or guilt?			
	Total	796	502	631
	Always	1 (0.1)	1 (0.2)	3 (0.5)
	Usually	9 (1.1)	7 (1.4)	11 (1.7)
	Sometimes	25 (3.1)	40 (8.0)	54 (8.6)
	Seldom	75 (9.4)	76 (15.1)	111 (17.6)
	Never	686 (86.2)	378 (75.3)	452 (71.6)
10	Does your partner have a problem with erections that affect your sexual activity?			
	Total	795	502	629
	Always	.	1 (0.2)	.
	Usually	2 (0.3)	10 (2.0)	2 (0.3)
	Sometimes	25 (3.1)	38 (7.6)	14 (2.2)
	Seldom	67 (8.4)	453 (90.2)	53 (8.4)
	Never	701 (88.2)		560 (89.0)
11	Does your partner have a problem with premature ejaculation that affect your sexual activity?			
	Total	796	502	629
	Always	2 (0.3)	3 (0.6)	3 (0.5)
	Usually	11 (1.4)	10 (2.0)	21 (3.3)
	Sometimes	56 (7.0)	38 (7.6)	50 (8.0)
	Seldom	167 (21.0)	85 (16.9)	117 (18.6)
	Never	560 (70.4)	366 (72.9)	438 (69.6)
12	Compared to orgasms you have had in the past, how intense are the orgasms you have had in the past 6 months?			
	Total	788	496	628
	Much more intense	15 (1.9)	9 (1.8)	11 (1.8)
	More intense	114 (14.5)	76 (15.3)	88 (14.0)
	Same intensity	600 (76.1)	336 (67.7)	425 (67.7)
	Less intense	51 (6.5)	63 (12.7)	84 (13.4)
	Much less intense	8 (1.0)	12 (2.4)	20 (3.2)

PISQ-12 answers were not calculated at 8 weeks postpartum, since PISQ-12 requests the participant to consider the last 6 months

## Abbreviations

OASI: Obstetric anal sphincter injury
PISQ-12: Pelvic Organ Prolapse/Urinary Incontinence Sexual Questionnaire of 12 questions
PISQ-IR: Pelvic Organ Prolapse/Urinary Incontinence Sexual Questionnaire IUGA-Revised
POPRACT: Pelvic flOor in PRegnancy And ChildbirTh study
